# A Review on Fluoroquinolones’ Toxicity to Freshwater Organisms and a Risk Assessment

**DOI:** 10.3390/jox14020042

**Published:** 2024-06-04

**Authors:** Marianna Pauletto, Marco De Liguoro

**Affiliations:** Department of Comparative Biomedicine & Food Science (BCA), University of Padova, Viale dell’Università 16, I-35020 Legnaro, Padova, Italy; marianna.pauletto@unipd.it

**Keywords:** fluoroquinolones, antibiotics, EC_50_, PNEC, freshwater ecosystem, normalized Species Sensitivity Distribution

## Abstract

Fluoroquinolones (FQs) have achieved significant success in both human and veterinary medicine. However, regulatory authorities have recommended limiting their use, firstly because they can have disabling side effects; secondly, because of the need to limit the spread of antibiotic resistance. This review addresses another concerning consequence of the excessive use of FQs: the freshwater environments contamination and the impact on non-target organisms. Here, an overview of the highest concentrations found in Europe, Asia, and the USA is provided, the sensitivity of various taxa is presented through a comparison of the lowest EC_50s_ from about a hundred acute toxicity tests, and primary mechanisms of FQ toxicity are described. A risk assessment is conducted based on the estimation of the Predicted No Effect Concentration (PNEC). This is calculated traditionally and, in a more contemporary manner, by constructing a normalized Species Sensitivity Distribution curve. The lowest individual HC5 (6.52 µg L^−1^) was obtained for levofloxacin, followed by ciprofloxacin (7.51 µg L^−1^), sarafloxacin and clinafloxacin (12.23 µg L^−1^), and ofloxacin (17.12 µg L^−1^). By comparing the calculated PNEC with detected concentrations, it is evident that the risk cannot be denied: the potential impact of FQs on freshwater ecosystems is a further reason to minimize their use.

## 1. Introduction

During the 1970s, a groundbreaking development emerged in the realm of pharmaceuticals with the introduction of flumequine. Scientists successfully enhanced the quinolone structure by incorporating a fluorine atom at position C6 [1]. This chemical modification not only broadened the spectrum of antimicrobial activity but also significantly improved tissue penetration. The subsequent pursuit of innovation led to the discovery of numerous other fluoroquinolones (FQs) that surpassed the efficacy of flumequine in terms of therapeutic range and pharmacokinetic properties in both humans and animals. Despite initial successes, certain FQs, including alatrofloxacin, clinafloxacin, gatifloxacin, gemifloxacin, grepafloxacin, sparfloxacin, temafloxacin, and trovafloxacin, faced swift withdrawal from the market due to severe adverse reactions [2,3]. However, other FQs have progressively emerged as the preferred choice for treating serious infections and common ailments, even though they have occasionally shown alarming side effects.

Over time, the pharmaceutical landscape evolved, and today, a diverse array of FQs exists, categorized into first, second, third, and fourth generations [4]. The development of new FQs has made an important contribution to the advancement of human medicine, with compounds such as ciprofloxacin, delafloxacin, gemifloxacin, levofloxacin, lomefloxacin, moxifloxacin, ofloxacin, pefloxacin, prulifloxacin, and rufloxacin routinely being used in healthcare settings. Additionally, veterinary medicine has benefited from dedicated FQs, such as flumequine, enrofloxacin, danofloxacin, pradofloxacin, difloxacin, marbofloxacin, sarafloxacin, and orbifloxacin [5,6].

FQs exert their antimicrobial effects by selectively inhibiting two bacterial enzymes, DNA gyrase and topoisomerase IV, which are crucial for bacterial DNA replication but absent in human cells [7]. This targeted mechanism would have made FQs specific and bactericidal, offering promising therapeutic prospects; however, research has revealed that DNA gyrases are not exclusive to bacteria, as green algae [8] and higher plants [9] also possess these enzymes. Furthermore, recent studies have shown that ciprofloxacin can interfere with type II topoisomerases (Top2α and Top2β) found in mammalian mitochondria, leading to mitochondrial damage, a phenomenon referred to as mitotoxicity [10]. Mitochondrial disruption has been associated with delayed adverse effects ranging from neurologic to musculoskeletal and cardiovascular conditions [11]. Moreover, FQs have been implicated in iron chelation, resulting in the inhibition of Fe (II)-dependent dioxygenases. This inhibition leads to DNA and histone hypermethylation, suppression of collagen prolyl hydroxylation, and inhibition of HIF mRNA translation. These molecular alterations may explain side effects, such as renal toxicity and tendinopathy [12].

As the usage of FQs in human medicine gained momentum, concerns about side effects grew. In 2015, the FDA (U.S. Food and Drug Administration) recognized FQAD (FluoroQuinolone-Associated Disability) as a syndrome, based on 178 cases where otherwise healthy individuals developed disabling and potentially irreversible conditions after taking fluoroquinolones for minor ailments. FQAD involves various symptoms that may endure even after stopping the medication. These symptoms might consist of profound tiredness, muscle frailty, joint discomfort, nerve impairment, cognitive challenges, and disturbances in mental health. Consequently, the FDA revised the boxed warning on FQs to address safety concerns [13] and advised reserving these drugs for serious infections only [2]. Similarly, the European Medicines Agency’s Pharmacovigilance Risk Assessment Committee (PRAC) recommended restricting the use of FQs and quinolone antibiotics in 2018, following reports of disabling and potentially long-lasting side effects associated with these medications [14].

In veterinary medicine, the use of FQs, particularly enrofloxacin, has also raised concerns due to reported side effects on domestic animals, affecting the skeletal and reproductive system, retinal pigment epithelium, liver enzymatic activity, and immune system [11]. These findings, combined with the risk of spreading antibacterial resistance between animals and humans, have discouraged the widespread use of FQs in both human and veterinary medicine [12]. Consequently, the once-growing trend of FQ utilization in human medicine has ended, and there is now a greater focus on judicious and restricted administration. However, although there were some modest reductions in prescriptions after the implementation of interventions in some countries, findings did not support a relevant effect of regulatory intervention on FQ use [15].

For widely used drugs like FQs, which find application in both human medicine and farm animals and are not strictly selective in their action, the potential impact on the environment has become a critical concern. FQs are known for their remarkable persistence [16], and recent scientific literature has shed light on their presence in the environment and their toxic effects, particularly on aquatic organisms [17,18]. Studies have revealed that FQs can harm both photosynthetic organisms and animals, even at low environmental concentrations. Indeed, they bear the potential to affect biodiversity through their impacts on sensitive species. Moreover, the ability of these compounds to interact with genetic material and induce delayed, reproductive, and transgenerational toxicity [19,20,21] adds to the complexity of assessing their environmental risk.

In light of these concerns, the primary objective of this paper is to provide a comprehensive and critical review of the existing knowledge regarding the presence of FQs in the aquatic environment and their toxic effects on freshwater organisms. For this purpose, a database on drug concentrations in the environment [22] was used as the main reference point for environmental contamination, whilst published data on toxic effects in freshwater organisms were retrieved based on the U.S. Environmental Protection Agency (USEPA) ecotoxicological database [23], the supplementary material of a recent review on FQs [24], and with the help of various search engines, by using the keywords “fluoroquinolones, environment, freshwater, ecotoxicity” to retrieve the relevant literature. The main criteria for selecting articles on contamination levels were the precise location of the sampling and validation of the analytical method reported; whilst for articles on toxic effects, the availability of accurate measures of acute toxicity (EC_50_) and/or a clear description of the mechanisms involved were discriminative. By consolidating the available information, this review aims to enhance our understanding of the potential risks posed by FQs and their impact on delicate freshwater ecosystems. Of the numerous FQs, only 11 (ciprofloxacin, clinafloxacin, enrofloxacin, flumequine, gatifloxacin, levofloxacin, lomefloxacin, moxifloxacin, norfloxacin, ofloxacin, and sarafloxacin) were considered for risk assessment, taking into account the availability of data on both their presence in the environment and toxicity to freshwater organisms. Recently, various reviews have been published on FQs in the environment [24,25,26,27,28,29]. This one may complement them by focusing on the freshwater ecosystem, giving a broad picture of the historical contamination (first quarter of the current century) in Europe, Asia, and the USA, accurately updating the mechanisms of toxicity in non-target species, and performing a novel risk assessment based on a normalized Species Sensitivity Distribution.

## 2. Chemistry and Mode of Action of FQs

Flumequine was the first mono-fluorinated quinolone. The addition of fluorine, in C6, to the basic quinolone structure, resulted in an increased antibacterial spectrum. This marked the beginning of intensive chemical synthesis efforts to refine structure–activity relationships and optimize pharmacokinetics. The addition of different R1, R7, and R8 groups created new and more effective FQs with broader antibacterial spectra and improved pharmacokinetics. A detailed table on the structure of the various FQs is reported in [30]. Each generation of FQs has specific advantages and disadvantages, including differences in the spectrum of activity, pharmacokinetics, side effects, and resistance patterns. According to Rusu and colleagues [31], there are four distinct generations of FQs. Of the compounds considered here for the risk assessment, flumequine belongs to the first generation; ciprofloxacin, lomefloxacin, norfloxacin, ofloxacin, and sarafloxacin to the second; clinafloxacin, enrofloxacin, and levofloxacin to the third; gatifloxacin and moxifloxacin to the fourth. FQs are used to treat a wide range of bacterial infections mainly of the urinary and respiratory tract, and are often used in cases where other antibiotics are not suitable due to resistance or allergies.

The targets of FQs are two enzymes that regulate DNA topology: gyrase and topoisomerase IV. Each of these enzymes is composed of four subunits: two GyrA and two GyrB make up the gyrase, two ParC and two ParE the topoisomerase IV. The GyrA and ParC are involved in DNA strand breakage, whilst GyrB and ParE in DNA strand assembling. Through its breaking and re-ligation processes, DNA gyrase alleviates supercoiling ahead of the replication fork, paving the way for the replication complexes to move along the DNA, whilst Topoisomerase IV, at the end of replication, removes the tangling of DNA strands, thereby allowing DNA segregation into two daughter cells. Fluoroquinolones interact with the DNA-bound enzyme to create conformational changes that result in the inhibition of normal enzyme activity. As a result, in the case of gyrase, the progression of the replication fork is blocked, thereby inhibiting normal bacterial DNA synthesis, whilst in the case of topoisomerase IV, the bacterial circular DNA is broken but no longer re-ligated [7].

## 3. Sources and Presence of FQs in the Environment

Wastewater from aquaculture facilities, pharmaceutical manufacturing suites, hospitals, and municipalities are major sources of watersheds contaminated with FQs. Traditional wastewater treatment plants are ineffective in removing fluoroquinolones [27]. By comparing levels in influents and effluents of 18 WWTPs around the world [32], an average removal efficiency of 64 ± 32% can be calculated for the various FQs. Additionally, a significant quota of FQs may end up in dewatered sludge [33], which is sometimes used as fertilizer, representing an additional input route into the environment. In addition, manure/slurry from large-scale animal farms where FQs are used for mass medication (prophylactic or metaphylactic) are often used as fertilizer for crop fields, contributing greatly to soil contamination [34]. Once brought onto land, FQs tend to sorb strongly at the topsoil [35]. This phenomenon prevents leaching to lower soil layers and groundwater contamination [36]; however, FQs can still reach surface water bodies through agricultural runoff [29].

**Figure 1:**
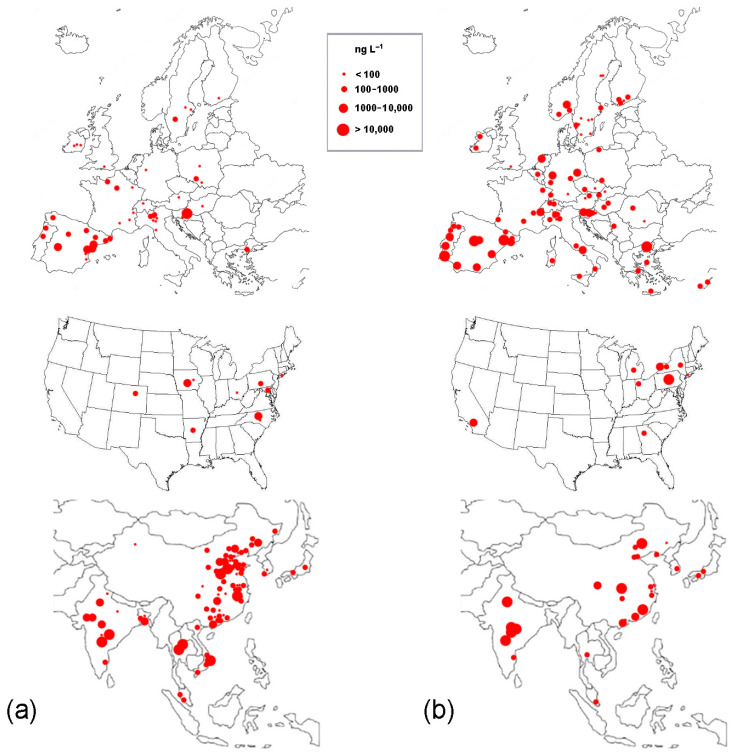
Highest levels of fluoroquinolones detected in surface waters (**a**) and WWTP effluents (**b**) of Europe, the USA, and Asia. The red spot size indicates the concentration range (see inset). Data are obtained from [24,25,33,40,41,42,46,48,49,50,51,52,53,54,55,56,57,58,59,60,61,62,63,64,65,66,67,68,69,70,71,72,73,74,75,76,77,78,79,80,81,82,83,84,85,86,87,88,89,90,91,92,93,94,95,96,97,98,99,100,101,102,103,104,105,106,107,108,109,110,111,112,113,114,115,116,117,118,119,120,121,122,123,124,125,126,127,128,129,130,131,132,133,134,135,136,137,138,139,140,141,142,143,144,145,146,147,148,149,150,151,152,153,154,155,156,157,158,159,160,161,162,163,164,165,166,167,168,169,170,171,172,173,174,175,176,177,178,179,180,181,182,183,184,185,186,187,188,189,190,191,192,193,194,195,196,197,198,199,200,201,202,203,204,205,206,207,208,209,210,211,212,213,214,215,216,217,218].

In general, the adsorption of antibiotics to soils is determined by organic content, pH, cation exchange capacity, and soil texture [37]. Despite a relatively low octanol–water partition coefficient (K*_ow_*), the various FQs show a high affinity for sludge, soils, and sediments. Indeed, depending on the pH, they occur as a mixture of neutral and/or differently charged species, and electrostatic interactions may play a significant role in the sorption process [30]. FQs are known to have moderate-to-high persistence in soils and sediments, which can lead to their accumulation over time [27]. Indeed, concentrations up to a few mg kg^−1^ have been detected in freshwater sediments [38,39], soils fertilized with contaminated manure [34], and Waste Water Treatment Plants (WWTPs) dewatered sludge [33]. Concentrations detected in the water column have been generally in the ng to µg L^−1^ range [40,41], with some remarkable exceptions at the mg L^−1^ level, resulting from drug manufacturing activities [42,43,44] and fish farming [45] in Asian countries. In the area of the Pearl River delta (China), traces of FQs have been detected even in tap water [46,47]. In Figure 1, the highest concentrations detected in surface waters or WWTP effluents of the various countries are considered and represented by spots of different sizes according to the range they fall into.

## 4. Mechanisms of Toxicity of Fluoroquinolones in Non-Target Species

Once FQs have been released into the aquatic environment, they interact with the microbial communities, leading to changes in their diversity, abundance, and function. Indeed, FQs inhibit the growth of both susceptible pathogenic and beneficial bacteria, resulting in the disruption of microbial ecosystems (e.g., shift in nutrient cycling, alteration of decomposition rates) peculiar to freshwater environments [27]. In addition to this general effect, common to all antibiotics, FQs can induce a plethora of toxic effects through specific molecular mechanisms, some of which have been largely studied, whilst others have poorly so, and some have been only postulated [3]. In the present section, toxic effects and their putative molecular mechanisms of particular relevance to the freshwater species are reported. Worthy of note, molecular mechanisms have been originally investigated in mammals, and only in some cases in fish and crustaceans, but rarely studied in microalgae and aquatic plants. Thus, what we know about FQ mechanisms of toxicity mostly results from studies conducted in mammalian cells and rodents, and marginally in zebrafish and daphnids. Overall, whilst the phenotypic effects caused by FQs have been extensively studied across various freshwater species, their correlation with distinct molecular mechanisms remains inadequately explored. In this respect, mechanistic studies specifically assessing the molecular drivers of FQ toxicity should be encouraged.

### 4.1. Genotoxicity

The same mechanisms of action underpinning antibacterial effects of FQs might be responsible for toxicity in non-target eukaryotic organisms. Indeed, whilst initially FQs were deemed to specifically target prokaryote gyrase and topoisomerases, later on, it was discovered that enzymes with similar structures and functions might occur also in eukaryotic cells (green algae, plants, and mammals). This is the case for type II topoisomerases Top2α and Top2β, recently identified in mammalian mitochondria extracted from cultured cells and mouse tissues [10]. In particular, ciprofloxacin showed the ability to interact with the DNA-bound mammalian Top2β, thus inhibiting the enzyme activity, and resulting in the accumulation of positively supercoiled mtDNA, blockage of mitochondrial transcription and replication, reduction in mtDNA copy number, and, ultimately, impaired cell proliferation [10]. Likewise, proteomic investigations conducted in vivo in zebrafish embryos showed that ciprofloxacin and enrofloxacin decreased Top1 levels [219], most probably as a consequence of the binding between FQs and the topoisomerase, leading to enzyme degradation.

Mutagenic effects can also be caused by non-selective DNA binding of FQs that induces oxidative DNA damage (ROS overproduction), in turn causing cell death and/or accelerating cellular aging, as previously demonstrated for several drugs [220,221]. Indeed, in a recent study, the genotoxic effects of five of the FQs most used in human medicine (ciprofloxacin, levofloxacin, moxifloxacin, norfloxacin, and ofloxacin) were demonstrated through in vitro and in silico combined approaches [222]. The authors showed a discrete ability of these FQs to bind human DNA, resulting in oxidative damage and base excision repair (BER) pathway activation.

Genotoxic effects of FQs were also highlighted in vivo. In *Drosophila melanogaster*, ciprofloxacin and enrofloxacin were found to induce homologous recombination in proliferative cells, a DNA repair mechanism that can result in DNA rearrangements and genetic diseases [223]. In this study, homologous recombination is suggested to occur as a consequence of DNA double-strand breaks not resolved because of FQ-mediated inhibition of Top2, which affects DNA breakage/rejoining reactions. In the liver of yellow catfish *Pelteobagrus fulvidraco*, enrofloxacin significantly increased the level of GADD45, a protein frequently induced by DNA damage, and promoted oxidative stress and apoptosis [224]. Chronic exposure to ciprofloxacin induced genetic damage in *D. magna*, yet in this study oxidative stress occurred marginally, namely with a mild increase in lipid peroxidation observed [225]. Likewise, environmental concentrations of ciprofloxacin and enrofloxacin induced cellular DNA damage (i.e., DNA strand breaks) and apoptosis in zebrafish embryos, but only partly due to increased ROS [219]. Indeed, in addition to oxidative stress, other mechanisms may also contribute to DNA damage, such as the inhibition of topoisomerases, as discussed above, and the inhibition of the DNA repair machinery [219].

It is well established that the chemical structure of FQs is closely related to their genotoxic potential. Firstly, the fluorine atom in C6 improves their antibacterial efficacy but, unfortunately, also increases the genotoxicity of the compounds [226,227]. Secondly, QSAR and 3D Pharmacophore in silico models showed that the C5 and C7 positions are the main determinants of FQ genotoxicity [228]. It is worthy of note that new FQ derivatives, based on this prediction model, present lower genotoxicity and higher efficacy [228].

### 4.2. Degradation of the Extracellular Matrix

A further mechanism underlying FQ toxicity is the upregulation of cell matrix metalloproteinases (MMPs), sometimes coupled to the downregulation of their inhibitors (tissue inhibitor of MMPs, TIMPs). The imbalance between the production of MMPs and TIMPs leads to the degradation of collagen and elastic fibers, thus affecting the integrity of the extracellular matrix (ECM) in several tissues [229,230]. ECM disruption likely serves as a mechanism for several adverse events. For instance, in an epidemiological study conducted by the Food and Drug Administration (FDA) and the European Medicines Agency (EMA), ciprofloxacin, levofloxacin, and moxifloxacin administration were associated with potential aortic dissections or rupture [231]. Ciprofloxacin-mediated tympanic membrane healing problems might be due to a decrease in fibroblast viability and collagen and α-tubulin protein levels [232]. Likewise, ciprofloxacin and levofloxacin decreased the principal matrix protein collagen type I in human-derived tendon cells, possibly resulting in tendinopathies [233]. In addition, FQs possess chelating properties. For instance, ciprofloxacin, enrofloxacin, and norfloxacin strongly chelate the iron needed by the two enzymes crucial for collagen synthesis, prolyl 4-hydroxylase and lysyl hydroxylase, as demonstrated in human embryonic kidney cells [12]. Furthermore, it is possible that FQs may disrupt the ECM outside the retina, a tissue that contains several layers of different kinds of collagen, possibly resulting in retinal detachment. Despite this adverse effect remaining controversial, some epidemiological studies reported an association between oral administration of FQs and the risk of developing retinal detachment (e.g., [234,235]).

This mechanism of FQ toxicity which targets the ECM is poorly studied in aquatic species. Environmental concentrations of norfloxacin have recently been reported to affect zebrafish craniofacial development, putatively dysregulating the expression of genes coding for ECM proteins (e.g., collagen type II) [236]. In embryos of the same species, enrofloxacin and ciprofloxacin ≤ 0.5 μM modulate the protein level of major ECM components, namely collagens, fibronectin, and laminin [237]. Overall, based on evidence in fish and humans, we would infer that the FQ-induced dysregulation of ECM proteins might cause structural defects that may ultimately result in alterations of many tissues, with serious effects on the freshwater biota.

### 4.3. Toxicity to the Central Nervous System

FQs are antagonists of Gamma-Aminobutyric Acid-A (GABA-A) receptors in the brain, spinal cord, and peripheral nervous system, meaning that they displace other molecules that physiologically bind to this receptor, such as GABA. As it is an inhibitory neurotransmitter, FQs induce blockage of GABA receptors, which results in a stimulation of the central nervous system (CNS), with neurological manifestations encompassing insomnia, agitation, hallucinations, and seizures in humans. Difference in FQs’ affinity for GABA-A receptors most likely explains the variability in neurotoxic effects. Indeed, FQs containing unsubstituted 7-piperazine (e.g., ciprofloxacin, enoxacin, norfloxacin) and 7-pyrrolidine (e.g., clinafloxacin, tosufloxaxin) at their R7 position show the most significant GABA-antagonistic effects [238]. Accordingly, in zebrafish, FQs were reported to affect locomotor activity, which is a good proxy of toxicity to CNS, and a correlation between FQ structure and the exerted toxicity was also highlighted [239].

FQs may also exhibit a direct effect on excitatory brain pathways, by stimulating N-methyl-d-aspartate (NMDA) and adenosine receptors, which could lead to other CNS symptoms [240,241]. A recent study has demonstrated the occurrence of this mechanism of toxicity in zebrafish embryos exposed to FQs [242]; in the aforementioned study, developmental neurotoxicity induced by norfloxacin was demonstrated to be mediated by the activation of NMDA receptors. The involvement of these receptors in mediating FQ toxicity has been reported also in *D. magna* exposed to flumequine, where NMDA type I was among the top upregulated genes [19].

## 5. Toxicity of FQs to Freshwater Organisms

For the convenience of the reader, in Table 1, all the collected EC_50s_ of FQs in cyanobacteria, unicellular algae, plants, crustaceans, and fish are reported and referenced, with details on duration, endpoints, and measured parameters.

### 5.1. Cyanobacteria

Enrofloxacin and ciprofloxacin were assayed on the cyanobacterium *Anabaena flos-aquae*, which was shown to be particularly sensitive, with EC_50_ values of 173 and 10.2 µg L^−1^, respectively [243]. In the same species, Gonzalez-Pleiter and co-authors reported an EC_50_ of 5.6 and 4.8 mg L^−1^ for norfloxacin and levofloxacin, respectively [244]. Robinson et al. [245] evaluated the toxicity of seven FQs on the cyanobacterium *Microcystis aeruginosa*; the most active compound was levofloxacin (EC_50_ 7.9 µg L^−1^), whilst the least was flumequine (EC_50_ 1.96 mg L^−1^). However, in the same species, Lützhøft and colleagues reported EC_50s_ of 15 µg L^−1^ for sarafloxacin and 159 µg L^−1^ for flumequine [247]; whilst Halling-Sorensen and colleagues reported an EC_50_ of 5 µg L^−1^ for ciprofloxacin [246]. In another study, it was found that the effects of norfloxacin on *M. aeruginosa* were modulated by light, the lowest EC_50_ being around 45 µg L^−1^ at high light intensity [249]. Ciprofloxacin was very toxic also to *Microcystis panniformis*, with an EC_50_ of 13.56 µg L^−1^ [171]. The EC_50_ values of gatifloxacin and moxifloxacin measured in *Chlamydomonas reinhardtii*, over 4–12 days of culturing, decreased progressively from 12.65 to 5.51 mg L^−1^ and from 83.04 to 17.39 mg L^−1^, respectively [261]. Their toxicity to *M. aeruginosa* was about three orders of magnitude greater, with 96 h EC_50_ of 25.30 and 60.34 µg L^−1^, respectively [248]. In summary, FQs are highly toxic to cyanobacteria since, in the majority of cases, their EC_50_ is lower than 1 mg L^−1^. This is not surprising as they are designed to act against prokaryotes. However, only a few classes of antibiotics (i.e., Aminoglycosides, Beta-lactams, Macrolides) have shown a comparable degree of toxicity toward cyanobacteria [280].

### 5.2. Unicellular Green Algae

Many authors have published data on the toxicity of FQs to *Raphidocelis subcapitata*, formerly known as *Selenastrum capricornutum*. In this green alga, Aderemi and co-authors evaluated the effects of ciprofloxacin and found a Lowest Observed Effect Concentration (LOEC) for growth inhibition of 6.329 mg L^−1^, whilst the most sensitive physiological endpoint among the various ones considered (superoxide dismutase activity, lipid peroxidation level, total energy content, energy consumption, and cellular energy allocation) was increased energy consumption (LOEC = 3.810 mg L^−1^), which was interpreted as the consequence of the need to respond to oxidative stress [281]. Lower concentrations of ciprofloxacin, corresponding to those measured in hospital wastewater (range 0.01–100 µg L^−1^), were assayed on *R. subcapitata* by Mater and colleagues [282], who found no effect on growth; however, when ciprofloxacin was mixed with analogous concentrations of tamoxifen or tamoxifen plus cyclophosphamide, a synergistic effect was evidenced, even at the lowest level assayed (0.01 µg L^−1^ of each compound). Liu and colleagues investigated the effects of ciprofloxacin on the photosynthetic process of *R. subcapitata* by determining various parameters, including photosynthetic rate, chlorophyll fluorescence, Hill Reaction, and ribulose-1.5-bisphosphate carboxylase activity [283]. They observed a significant reduction in the photosynthetic activity under concentrations ≥ 1 mg L^−1^. On the same green alga, Magdaleno et al. [250] assayed the growth inhibition activity of six compounds pertaining to different classes of antibacterials and found that ciprofloxacin was the most toxic, with an EC_50_ of 11.3 mg L^−1^, and an EC_10_ of 3.3 mg L^−1^. Once again, ciprofloxacin showed synergistic effects when assayed in binary mixtures (ratio 1:1) with gentamicin or vancomycin. The growth inhibition of ciprofloxacin in *R. subcapitata* was studied also in [246,251], who reported EC_50s_ of 2.97 and 4.83 mg L^−1^, respectively. Robinson et al. assayed the toxicity of seven FQs on various aquatic organisms [245]; clinafloxacin was the most toxic to *R. subcapitata*, with an EC_50_ of 1.1 mg L^−1^. The toxicity of norfloxacin and levofloxacin to *R. subcapitata* was investigated by Gonzalez-Pleiter and colleagues [244]; they observed only moderate effects on algal growth at the highest assayed concentration; both norfloxacin (120 mg L^−1^) and levofloxacin (80 mg L^−1^) caused less than 50% inhibition. However, the two compounds showed synergistic interaction in binary mixtures when combined together or with other antibiotics such as erytromicin and tetracyclin. The results of Yamashita et al. regarding levofloxacin toxicity were quite different, with an EC_50_ of 1.2 mg L^−1^, an LOEC of 0.360 mg L^−1^, and a total inhibition of *R. subcapitata* growth at concentrations exceeding 2.5 mg L^−1^ [257]. For the same endpoint, Yang et al. reported an LOEC of 16 mg L^−1^ for norfloxacin and 5 mg L^−1^ for ciprofloxacin [252]. Eguchi et al. estimated norfloxacin EC_50_ values of 16.6 and 10.4 mg L^−1^ in the green algae *R. subcapitata* and *Chlorella vulgaris*, respectively [258]. Fu et al. evaluated the toxicity of three FQs to *R. subcapitata* [253]; their calculated growth inhibition EC_50s_ after 96 h of exposure correspond to 7.082 (ciprofloxacin), 59.404 (norfloxacin), and 4.241 mg L^−1^ (ofloxacin). Indeed, the high toxicity of ofloxacin to green algae had been previously evidenced by Isidori et al. and Ferrari et al. with reported EC_50s_ of 1.44 and 4.74 mg L^−1^, respectively [259,260]. Lützhøft et al. evaluated the toxicity to *R. subcapitata* of two FQs used in aquaculture, namely flumequine and sarafloxacin [247]; the reported EC_50s_ for growth inhibition were 5 and 16 mg L^−1^, respectively. The toxicity of flumequine toward *R. subcapitata* was evaluated also by Zounková et al. [254], Van Der Grinten et al. [255], and Christensen et al. [256]; their calculated EC_50s_ were in the range of 2.6–16 mg L^−1^.

Data regarding other algal organisms are also available. Backhaus et al. assayed various FQs on *Scenedesmus vacuolatus* [265]; flumequine was distinctly the most toxic, with an EC_50_ of 3.7 mg L^−1^. Andrieu et al. measured the toxicity of enrofloxacin and ciprofloxacin in *Chlorella* sp. [53]; the calculated EC_50s_ were 111 and 23 mg L^−1^, respectively. Ciprofloxacin toxicity to *C. vulgaris* was investigated also by [262,263], who reported an EC_50_ of 29.09 and 20.6 mg L^−1^ for growth inhibition, respectively. Both enrofloxacin and ciprofloxacin were assayed on the freshwater green alga *Desmodesmus subspicatus*: calculated EC_50s_ for growth inhibition were 5.568 and >8.042 mg L^−1^, respectively [243]. Enrofloxacin was assayed also on *Scenedesmus obliquus*, resulting in an EC_50_ of 45.10 mg L^−1^ after 72 h of exposure [266], whilst norfloxacin EC_50_ in the same species was 38.49 mg L^−1^ [267] or 50.18 mg L^−1^ [268]. Li and co-authors assayed moxifloxacin on *Chlorella sorokiniana* and *Scenedesmus dimorphus* [264]; their sensitivity was almost identical, with EC_50s_ of 28 and 26 mg L^−1^, respectively. In summary, the EC_50_ of FQs in unicellular algae ranges from 1 to 100 mg L^−1^, confirming that the toxicity of antibiotics for unicellular algae may be orders of magnitude lower than for cyanobacteria. It is worth noting, however, that in *P. subcapitata*, which is the most extensively tested species, EC_50s_ of less than 10 mg L^−1^ are found for many FQs.

### 5.3. Aquatic Plants

Among the different classes of antibiotics tested for phytotoxicity, FQs have generally proven to be the most potent class [284]. Ebert and colleagues evaluated the toxicity of enrofloxacin and ciprofloxacin on two freshwater macrophytes: the monocotyledonous *Lemna minor* and the dicotyledonous *Myriophyllum spicatum* [243]. *L. minor* showed to be far more sensitive to FQs than *M. spicatum*: EC_50s_ of enrofloxacin and ciprofloxacin were 107 and 62.5 µg L^−1^, respectively. Similar results with these two compounds had already been published by Robinson et al., who also assayed the toxicity of five other FQs: levofloxacin, clinafloxacin, lomefloxacin, ofloxacin, and flumequine [245]. All these compounds showed themselves to be distinctly toxic to *L. minor*, their EC_50s_ being in the range of 51–126 µg L^−1^, except for flumequine (EC_50_ 2470 µg L^−1^). Flumequine effects on the aquatic weed *Lythrum salicaria* were investigated by Migliore et al. [285]: a significant growth inhibition was observed only at 100 mg L^−1^, whilst low concentrations (50–500 µg L^−1^) induced hormesis on secondary roots. Nunes and co-authors showed the activation of the anti-oxidant defensive system in both *L. minor* and *Lemna gibba* after exposure to very low concentrations (range 5–195 µg L^−1^) of ciprofloxacin [286].

A study on mesocosm wetlands planted with *Phragmites australis* [287] revealed browning and eventual die-off of the plant because of exposure to 2 mg L^−1^ of ciprofloxacin for a period of 5 days. Brain and colleagues evaluated the toxicity of four FQs in *L. gibba*; the calculated EC_50s_ for wet mass reduction were the following: 97 µg L^−1^ of lomefloxacin, 185 µg L^−1^ of levofloxacin, 532 µg L^−1^ of ofloxacin, 698 µg L^−1^ of ciprofloxacin [269]. In the same species, the EC_50_ of norfloxacin was 913 µg L^−1^ [270]. In summary, the antibiotic FQs exhibit a toxicity to aquatic plants close to that observed for cyanobacteria and significantly higher than that exhibited towards unicellular algae. This is a crucial aspect of FQ ecotoxicity. Indeed, the presence and diversity of aquatic plants are essential for the health and functioning of aquatic ecosystems, providing numerous benefits to fish and crustaceans and other aquatic organisms by offering habitat, food, oxygen, refuge from predators, places for spawning and rearing young, and contributing to overall ecosystem stability and resilience.

### 5.4. Crustaceans

The toxicity of FQs to cladoceran crustaceans has been evaluated by various authors. Isidori and colleagues assayed the toxicity of ofloxacin in *D. magna* and *Ceriodaphnia dubia*. The latter was more sensitive to the antibacterial, with calculated EC_50s_ of 17.41 mg L^−1^ (48 h, immobilization test) and 3.13 mg L^−1^ (7-day, population growth inhibition) [259]. Using the Comet assay, Nunes et al. evidenced genotoxic effects in *D. magna* exposed to ciprofloxacin at a concentration ≥ 0.013 mg L^−1^, whilst they observed no effects in the life-history parameters of the crustacean up to 0.195 mg L^−1^ [225]. Kergaravat and co-authors measured the Lethal Concentration, 50% (LC_50_), of various FQs both in *D. magna* and *C. dubia* [275]. The authors showed that after 72 h of exposure, toxicity is about 3–4 times higher than after 48 h, with LC_50_ values <10 mg L^−1^ for the most active compounds (i.e., levofloxacin and moxifloxacin). They also reported results of the chronic reproduction test with the same two cladocerans; again, levofloxacin and moxifloxacin were the most toxic, with No Observed Effect Concentration (NOEC) values in *D. magna* of <2.5 and <1.6 mg L^−1^, respectively. Mala and Dutta evaluated the toxicity of gemifloxacin and gatifloxacin in *D. magna* [274]. Their EC_50s_ were two orders of magnitude greater than those of other FQs from previous generations, with values of 489.2 and 330.8 mg L^−1^, respectively. Eluk and co-authors evaluated both the 48 h EC_50_ and the 21-day NOEC of six FQs in *D. magna* [271]. The range of the estimated EC_50_ values was between 5.4 (marbofloxacin) and 36 mg L^−1^ (ofloxacin); the NOEC range was between 56 (ciprofloxacin) and 141 µg L^−1^ (ofloxacin). With regard to lomefloxacin, Luo et al. showed that exposure of *D. magna* under simulated sunlight radiation caused a remarkable increase in toxicity, with EC_50_ dropping from 166 to 64 mg L^−1^ [276]. Pan and co-authors evidenced effects on swimming activity and feeding rate in *D. magna* exposed to 25 mg L^−1^ of norfloxacin [288]. Dalla Bona and colleagues evaluated the acute toxicity of ciprofloxacin and enrofloxacin both in *D. magna* and *Daphnia curvirostris*: the latter showed to be more sensitive to the two FQs, with EC_50s_ of 4.45 and 4.33 mg L^−1^, respectively [273]. In both species, binary mixtures of the two compounds showed mainly sub-additive interaction. In multigenerational tests conducted in *D. magna*, enrofloxacin [289] and flumequine [20] exhibited intergenerational and transgenerational toxicity, respectively. It is worth noting that the observed phenotypic changes in daphnids did not impact all individuals within the specific experimental group. Rather, they manifested only in certain subjects, following a stochastic pattern that could potentially indicate an interaction of the two FQs with the genetic material. In the same freshwater organism, enrofloxacin, flumequine, and levofloxacin also showed the ability to induce delayed toxicity after neonatal exposure, with EC_50s_ recalculated after a follow-up of 10 days in pure medium, being 3.13, 7.18, and 15.11 mg L^−1^, respectively. Delayed toxicity was also observed after embryonic exposure of the crustacean to the three FQs, whilst their interaction in binary and ternary mixtures was merely additive [21]. Pietropoli and co-authors assessed the developmental and reproductive impairment caused by flumequine in *D. magna*, using an in vivo transcriptomic approach [19]. The lowest assayed concentration (0.2 mg L^−1^) had no noticeable impact on phenotypic traits; however, it did affect gene expression, and this effect became more pronounced at the highest exposure level (2 mg L^−1^), where phenotypic effects were evident and a significant modulation was detected in various genes associated with growth, development, structural components, and the antioxidant response. Dionisio and colleagues obtained an EC_50_ of 36.493 mg L^−1^ after acute exposure of *D. magna* to ciprofloxacin [272]; interestingly, they also showed that chronic exposure to relevant concentrations of ciprofloxacin may cause oxidative stress and that exposure of mothers to 3 mg L^−1^ of the compound may diminish the size of newborn daphnids. Ciprofloxacin and Enrofloxacin were assayed also on *Moina macrocopa;* their 48 h EC_50s_ were 71.2 and 69.1 mg L^−1^, respectively [53]. Zhang and co-authors evaluated the toxic effects of enrofloxacin in the giant freshwater prawn *Macrobrachium rosenbergii* [290]. One-week exposure of juvenile individuals to 1–5 mg L^−1^ of the pharmaceutical caused dose-dependent growth inhibition, whilst a concentration of 0.2 mg L^−1^ had growth-promoting effects. Instead, no toxic effects were observed in *Thamnocephalus platyurus* exposed to 100 mg L^−1^ of levofloxacin [291]. In summary, the toxicity of FQs to freshwater crustaceans can be considered moderate, EC_50s_ being usually in the order of tens of mg L^−1^; however, their evidenced ability to interact with gene expression and cause delayed and transgenerational effects suggests a possible impact on crustacean populations, even after short exposure in the natural environment, that the standard acute immobilization test cannot predict [19,20,21,273,289,292,293].

### 5.5. Fish

The toxicity of various FQs has been assayed on zebrafish (*Danio rerio*) embryos and, for all the compounds, crucial toxic effects on development were reported but only at concentrations of hundreds of mg L^−1^ [277]. Similar findings were reported by [242,278] after exposing zebrafish embryos to norfloxacin and enrofloxacin, respectively. Exposure of zebrafish to high concentrations of gatifloxacin reduced heart rate and cardiac output and resulted in pericardial edema [294]. In the same species, flumequine was shown to be teratogenic at relatively low concentrations (4.8–77 mg L^−1^), with an EC_50_ for embryo mortality of ~40 mg L^−1^ [279]. Nogueira and co-authors reported that 96 h exposure to ciprofloxacin (0.005–0.488 µg L^−1^) induced an increase in AChE (acetylcholinesterase) activity and a decrease in CAT (catalase) activity in *D. rerio* embryos or larvae [295]. Interestingly, 10 mg L^−1^ of norfloxacin caused a significant serum increase in vitellogenin in male goldfish (*Carassius auratus*). Moreover, in the same species, higher levels of norfloxacin were able to induce concentration- and time-dependent DNA alterations [296]. In *Xiphophorus helleri*, it has been shown that norfloxacin, even at a concentration < 1 mg L^−1^, can have effects on hepatic gene expression of P450 isoforms, GST (glutathione S-transferase), and P-glycoprotein [297]. Early-stage zebrafish exposed to norfloxacin nicotinate exhibited increased transcriptional levels and activities of major antioxidant enzymes like SOD (superoxide dismutase), CAT, and GPX (glutathione peroxidase) [298]. In juvenile common carp, Zhao and colleagues showed oxidative stress, damage to the intestinal barrier function, and change in the expression of immune-related genes after chronic exposure to very low concentrations of norfloxacin [299]. Yang and co-authors reported that ofloxacin, at concentrations > 0.05 mg L^−1^, significantly inhibits AChE, promotes EROD (7-ethoxyresorufin-O-deethylase), and increases SOD activities in *C. auratus* [300]. In the fathead minnow (*Pimephales promelas*, 7-day early life stage) seven FQs (i.e., ciprofloxacin, clinafloxacin, enrofloxacin, flumequine, levofloxacin, lomefloxacin, and ofloxacin) have been tested for effects on survival and growth [245]. The compounds showed limited toxicity, with NOEC at 10 mg L^−1^, the only exception being clinafloxacin, with 100% lethality at 10 mg L^−1^ (NOEC = 2 mg L^−1^). Enrofloxacin (11 mg L^−1^) caused 80% mortality in medaka fish (*Orizia latipes*) after 30-day continuous post-hatching exposure [301]. However, acute exposure (96 h) up to 100 mg L^−1^ caused no effects in juveniles (10–14 days post hatch) of the same species [302]. Likewise, acute post-hatching exposure (96 h) to 100 mg L^−1^ levofloxacin did not cause any acute toxic effect [291]. Zhang and colleagues evaluated the toxicity of an equi-concentrations mixture of four FQs (i.e., ciprofloxacin, ofloxacin, norfloxacin, enrofloxacin) on *D. rerio* larvae and estimated an EC_50_ of 481.3 mg L^−1^, based on a malformation rate endpoint [303]. In summary, fish have been shown to be less sensitive than other aquatic organisms to the acute toxicity of FQs. However, the ability of FQs to interact with gene expression, interfere with embryo development, and increase serum vitellogenin once again suggests a possible impact on populations, even after short exposure in the natural environment.

### 5.6. Other Freshwater Organisms

Very few data are available concerning the toxicity of FQs towards other freshwater organisms. He and colleagues [304] evaluated the toxicity of norfloxacin and ofloxacin in young *Bellamya aeruginosa* snail. At concentrations up to 300 mg L^−1^, no lethal effects were observed. However, they obtained EC_50s_ values based on activity inhibition rate. This effect was concentration- and exposure-time-dependent. After 48 h exposure, the calculated EC_50s_ were 141.3 (norfloxacin) and 222.6 (ofloxacin) mg L^−1^. The scarce toxicity (EC_50_>100 mg L^−1^) to mollusks was confirmed as well with moxifloxacin in the bivalve *Lampsilis siliquoidea* [305]. A reproduction study exposing the snail *Potamopyrgus antipodarum* for 52 days to 0.8 μg L^−1^ ciprofloxacin elicited no effects [306]. Overall, and considering also data obtained on marine mollusks, almost 75% of the tested FQs demonstrated negligible toxicity to this taxon [24].

A sediment toxicity study investigated reproduction effects of ciprofloxacin in *Lumbriculus variegatus* and *Chironomus riparius* during 28 days of exposure. Both species were exposed to 0.25, 0.5, 1.0, 2.0, and 4.0 μg kg^−1^, which did not cause any significant effects [306].

Ciprofloxacin and levofloxacin were assayed on *Xenopus laevis* larvae [307], but the highest concentration of the two FQs (100 mg L^−1^) showed no effects on body length after 96 h exposure.

## 6. Risk Assessment

**Figure 2:**
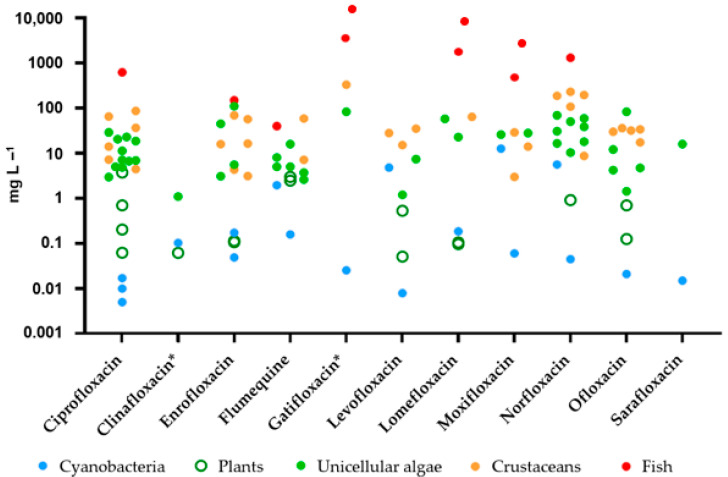
EC_50_ values (mg L^−1^) of fluoroquinolones in different freshwater taxa. Data are obtained from references listed in Table 1. * compounds that have been withdrawn from the market.

Overall, data indicate for freshwater organisms the following rank of sensitivity to FQs: cyanobacteria > aquatic plants > unicellular algae > crustaceans > fish. In Figure 2, to give an overview of the susceptibility of the aquatic biota to the toxicity of the various FQs, EC_50_ values obtained in the various taxa are presented.

When data are available for a reasonable number of species, a risk evaluation can be performed using the methodology outlined in [308]. Accordingly, results from eleven distinct FQ compounds across twelve diverse autotroph species were amalgamated in a Species Sensitivity Distribution (SSD) curve. By normalizing the acute toxicity values (EC_50s_) of each FQ to its EC_50_ in *M. aeruginosa*, the freshwater cyanobacterium highly sensitive to all FQs tested, the EC_50s_ were expressed as *Microcystis*-equivalents, enabling cross-FQs pooling. The set of geometric mean *Microcystis*-equivalent EC_50s_ (Table 2) was then analyzed using the USEPA SSD Generator V1, available at [309]. The endpoints considered to build the nSSD were: growth/yield inhibition (48 or 72 h) for cyanobacteria and unicellular algae, and seven-day growth/yield inhibition for plants. The decision to limit the nSSD curve to autotrophic species is motivated by the fact that they are more sensitive than heterotrophs to FQs and that, consequently, a safety threshold calculated for them can be a guarantee for all freshwater species.

**Figure 3:**
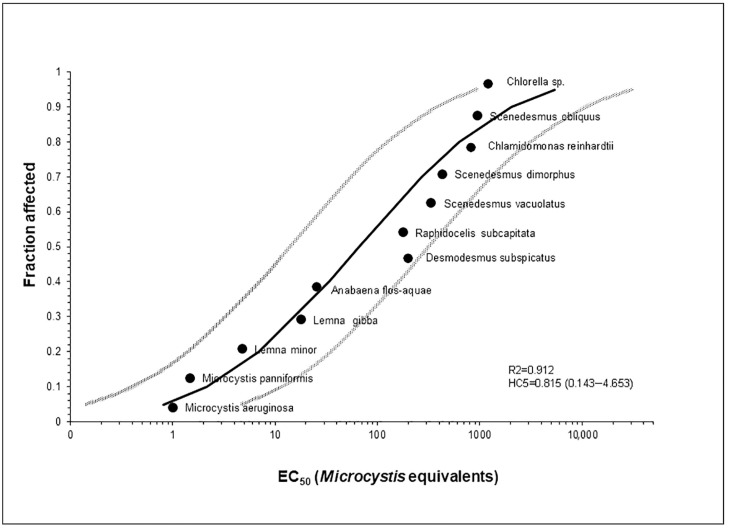
Species sensitivity distributions for twelve autotroph organisms, based on *Microcystis aeruginosa* equivalents for eleven fluoroquinolones; circles are equivalents for individual species, the solid line is model-fitted distribution, and greyed lines indicate 95% prediction interval.

In Figure 3, the *Microcystis*-equivalent SSD encompassing twelve species of autotroph organisms is illustrated, with prediction intervals and data points that led to the acquisition of the fifth percentile (Hazard Concentration, HC5), corresponding to 0.815, with a 95% confidence interval of 0.143–4.653. Multiplying this normalized value by the EC_50_ measured for each FQ in *M. aeruginosa*, the following individual HC5 values were then obtained: 7.51 µg L^−1^ (ciprofloxacin); 12.23 µg L^−1^ (clinafloxacin); 39.94 µg L^−1^ (enrofloxacin); 454.97 µg L^−1^ (flumequine); 20.62 µg L^−1^ (gatifloxacin); 6.52 µg L^−1^ (levofloxacin); 151.59 µg L^−1^ (lomefloxacin); 49.18 µg L^−1^ (moxifloxacin); 36.68 µg L^−1^ (norfloxacin); 17.12 µg L^−1^ (ofloxacin); 12.23 µg L^−1^ (sarafloxacin). These HC5 values may serve as effective metrics for preliminary risk assessments, as the comprehensive SSD can be harmonized with estimated exposure distributions for more comprehensive risk characterization [308].

**Figure 4:**
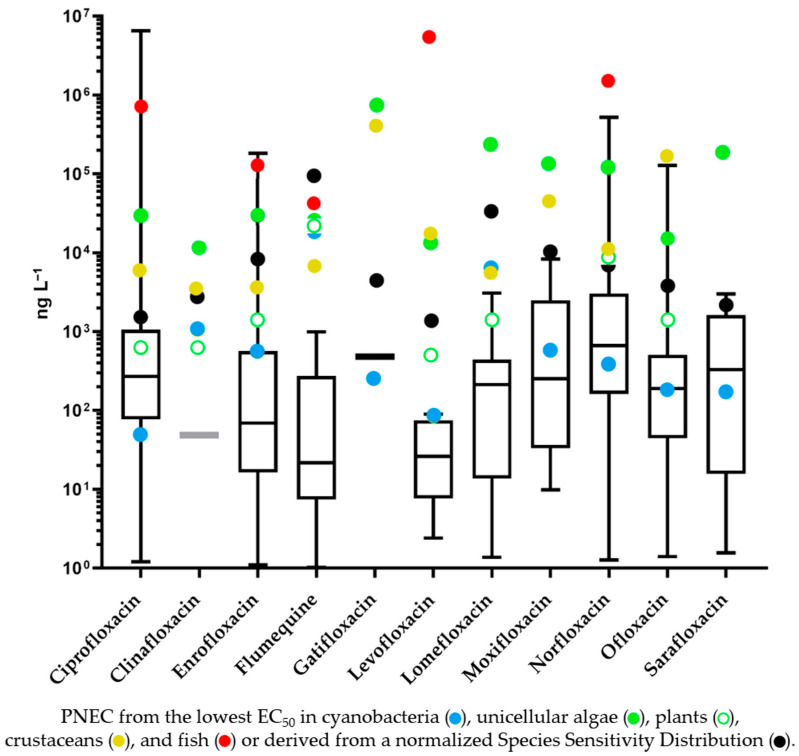
Box plot of the highest concentrations of fluoroquinolones detected in the freshwater environment compared with the PNEC calculated either in the traditional way, by applying a specific Assessment Factor to the lowest EC_50_ of cyanobacteria, unicellular algae, plants, crustaceans, and fish, or through a normalized Species Sensitivity Distribution in autotroph organisms (AF of 5 applied to the Hazard Concentration fifth percentile—HC5).

The normalized SSD for FQs provides a more comprehensive and statistically robust foundation for risk assessment in comparison to data from a single FQ in a single most sensitive species, which is instead advantageous for assessing compounds with data available for a limited number of species. Present-day risk assessment techniques incorporate an Assessment Factor (AF) that is adapted according to the data employed in SSD development and subsequently applied to the HC5 [311]. Ordinarily, it is recommended that the HC5 value be estimated from toxicity data including at least 4–8 species, and it is divided by a scaling AF ranging from 1 to 5 to determine the PNEC [312,313]. Even applying the most conservative AF of 5, the PNEC values calculated for the various FQs are higher, by about one order of magnitude, when compared to those obtained using an AF of 100 applied to the lowest EC_50_ in cyanobacteria (Figure 4). It is important to emphasize that the substantial AF (100) in the standard algorithm for single-compound risk evaluation primarily addresses the risk of overstating the PNEC in the face of extensive species sensitivity variations. Conversely, PNEC estimation through the SSD approach hinges predominantly on the number of species considered [312]. In the present analysis, twelve different species from the most sensitive taxa were included, suggesting an acceptable accuracy in PNEC determination, further supported by the high R2 value of the SSD curve (0.912).

Constructing an analogous SSD for heterotrophic organisms was hindered by data limitations, particularly concerning the absence of data for taxa such as mollusks and insects, along with sparse information available for amphibians and fish. The scarcity of data on fish results from their relatively lower sensitivity to FQs, leading to their infrequent use in acute toxicity tests. Moreover, the few available EC_50_ data for fish often fall into the “greater than” range, as it is not recommended to test concentrations above 100 mg L^−1^. Indeed, this concentration threshold is defined as a “limit test” by OECD Guideline 203 on “acute fish toxicity test” [314].

To allow for risk evaluation, in Figure 4, the PNEC, obtained either by applying an assessment factor (100 or 1000) to the lowest EC_50_ of each compound in the various freshwater taxa [315] or an assessment factor of 5 to the Hazard Concentration fifth percentile, is compared with the box plot of the highest freshwater concentration reported in the literature.

For the majority of compounds, the highest reported concentration is higher than the PNEC obtained by applying an assessment factor of 100 to the lowest EC_50_ in cyanobacteria. This is not surprising as the level of contamination in some specific areas of some countries in the Far East is extraordinarily high, mainly due to the activities of pharmaceutical companies and fish farms. Interestingly, with four FQs largely used in human medicine, namely ciprofloxacin, norfloxacin, ofloxacin, and sarafloxacin, even the median of the highest concentration detected in the aquatic environment is higher than the PNEC calculated in the traditional way; moreover, for two additional human pharmaceuticals (moxifloxacin and levofloxacin), the median is less than one order of magnitude lower than the PNEC. With regard to veterinary compounds, enrofloxacin, with a PNEC only an order of magnitude lower than the median, is still widely used in food-producing animals and could pose a risk to the aquatic environment in the years to come, particularly in countries where its use is also permitted in aquaculture. Notably, according to [316], six of the eleven largest aquaculture-producing countries currently use enrofloxacin.

With reference to the PNECs obtained from the nSSD curve, that, as we said, are one order of magnitude higher, there are still five FQs (ciprofloxacin, enrofloxacin, norfloxacin, ofloxacin, and sarafloxacin) with reported concentrations in the freshwater environment above the threshold. Consequently, a risk to the aquatic environment cannot be ruled out, particularly in those countries (India, China) where very high concentrations were most often detected. It should be noted, however, that an enormous effort has been made in China during the last decade in the search for drugs in the freshwater environment, and, as a consequence, a worse picture may have been obtained than in other countries. The risk regards autotroph organisms in particular and, considering their fundamental role in sustaining life of higher trophic level, bottom-up repercussions are inevitable. Indeed, they provide the aquatic fauna with not only food but also habitat, oxygen, and nutrient cycling. Macrophytes, in particular, can improve water quality by absorbing excess nutrients and pollutants, help stabilize sediments, provide refuge from predators and places for spawning and rearing young.

In a recent review from Thai and colleagues [25], regarding quinolone antibiotics in the Baiyangdian Lake (China), PNECs for flumequine and ofloxacin were presented, based on SSDs that included both autotrophic and heterotrophic species. They applied, like us, an AF of 5 and obtained PNEC values of 4.40 and 196 µg L^−1^ for ofloxacin and flumequine, respectively. These values are higher than those obtained by us (3.42 and 91 µg L^−1^). Of course, these differences could be justified by the exclusive use of autotroph organisms, the most sensitive taxa, to construct our nSSD curve.

Risk assessment should also consider the possible active environmental metabolites of FQs. These may be the result of enzymatic activity in the human [317] and the animal body [318,319] or the product of biotransformation by bacteria and fungi [320]. Photolysis products can also be active and, in some cases, even more toxic than the parent compound [321]. Unfortunately, comprehensive knowledge of all possible active metabolites of the various FQs is far from being achieved, partly because of the sophisticated techniques required for their identification and the limited availability of specific analytical standards for performing ecotoxicity assays.

Another aspect that should be considered in risk evaluation is the possible simultaneous presence of various FQs in a contaminated aquatic ecosystem. For mixtures of compounds having the same mechanism of action (MoA), the reference gold standard for predicting toxicological interaction is the Concentration Addition principle [322]. Accordingly, each chemical contribution to the overall toxicity of a mixture can be expressed as the quotient of its dose in the mixture and the dose of the same chemical alone that would be required to elicit the effect of the whole mixture. The few experiments carried out so far, on daphnids [21,273], indicate that binary and ternary mixtures of FQs display either additive or less-than-additive interaction. A similar conclusion was drawn by Backhaus et al. after assaying the toxicity of quinolone mixtures on *Vibrio fischeri* [323]. These findings are generally consistent with the data generated so far on the toxicity of chemical mixtures, as no interaction (simple additivity) or antagonism (less than additivity) has been observed in 95% of cases [324]. However, caution must be exercised when applying these results to the entire class of FQs: the fact that FQs share the same MoA does not necessarily imply that all potential combinations will not display synergistic interactions. For instance, Khadra et al. demonstrated a synergistic interaction in a ternary mixture composed of two FQs (ciprofloxacin and enrofloxacin) and one quinolone (nalidixic acid) when evaluating its genotoxicity in *Vicia faba* [325].

Overall, as autotrophs are far more sensitive than heterotrophs to FQ toxicity, a general safety threshold for FQs in freshwater could be assumed based on the HC5 dataset derived from the normalized SSD curve of autotrophs alone. Applying the most conservative AF (i.e., 5) to the lowest individual HC5 (6.52 µg L^−1^, levofloxacin), this safety threshold could be tentatively set at 1.30 µg L^−1^ (95% CI 0.23–7.44 µg L^−1^). Nonetheless, it would be advisable to determine such a threshold as the aggregate of all potential active ingredients stemming from FQ contamination within the freshwater ecosystem, metabolites included. Indeed, given that even low concentrations of single compounds may lead to severe overall toxicity when acting simultaneously on an organism, the toxicity of mixtures of pollutants has to be considered for the setting of environmental standards [323].

Finally, there is a lack of data on the toxicity of FQs to benthic fauna. This remains an important gap to fill, considering the tendency of FQs to accumulate in sediments. Indeed, the risk assessment presented here is based on the concentrations reported in the water column, which are generally one thousand times lower than those found in sediments [30]. In this regard, it should be emphasized that any damage to the biodiversity of freshwater benthic fauna can hinder the nutrient cycle that sustains life at all levels. Of course, benthic invertebrates play a crucial role by breaking down the organic detritus, mixing and aerating sediments, and promoting microbial growth [326].

## 7. Conclusions

Although FQs are recognized worldwide as “critically important antimicrobials”, they have been used indiscriminately in both human and veterinary health care in recent decades. Their limited metabolization and relative resistance to degradation have led to their widespread presence in municipal wastewater, as well as in manure/slurry, and to their consequent occurrence in various environmental compartments, including surface waters. In some specific areas, pharmaceutical production plants and aquaculture facilities have also contributed significantly to the contamination of watersheds. The short-term toxicity of FQs to freshwater organisms is remarkable, with EC_50s_ lower than 10 µg L^−1^ in cyanobacteria and 100 µg L^−1^ in aquatic plants. Indeed, for five FQs (ciprofloxacin, enrofloxacin, norfloxacin, ofloxacin, and sarafloxacin), the highest concentrations detected in the environment, when matched to the PNEC, give a Risk Quotient > 1. Moreover, FQs may induce delayed toxic effects and express embryonic and transgenerational toxicity in crustaceans: all features that are presumably related to their ability to interact with the genome of non-target organisms and may add complexity to risk assessment. Even in fish, which are far less sensitive than other aquatic organisms to the toxicity of FQs, toxic effects have been reported (e.g., genotoxicity, ECM degradation, neurotoxicity) and their putative underlying mechanisms postulated. Generally, our knowledge regarding the mechanisms of action in non-target species within freshwater environments is limited. Nevertheless, the existing information is already cause for concern.

More specifically, the use of this class of antimicrobials should be limited for many reasons:

1. Their particular efficacy in treating a range of dangerous bacterial infections should be safeguarded, limiting the spread of drug resistance as much as possible;
2. As with any class of antibiotics, limiting their use helps to maintain the ecological integrity of aquatic microbial communities;
3. The extent of their removal by conventional wastewater treatment plants is unsatisfactory;
4. Contamination of the freshwater environment is already widespread, and, in some cases, they achieve remarkably high levels;
5. Their relative resistance to degradation combined with a strong tendency to adsorption favors their progressive accumulation in sediments;
6. Their pronounced toxicity to organisms at basal trophic levels can have bottom-up repercussions in aquatic ecosystems;
7. Their potential ability to disrupt aquatic plant communities may influence the structure of the habitats provided by plants to various organisms;
8. Little is known about possible toxicological interactions amongst FQs or with other co-occurring contaminants of the freshwater environment;
9. Their possible transgenerational and delayed toxicity increases the complexity of defining environmental safety thresholds;
10. Given their ability to interact with the genome, even brief occasional exposure of freshwater organisms to FQs could have long-term consequences;
11. The restriction of FQ use aligns with broader efforts to promote sustainable water management practices, emphasizing the protection and conservation of aquatic ecosystems for future generations.

Overall, the toxicity to freshwater organisms of the eleven compounds considered here does not appear to vary significantly among FQ generations (Figure 2); however, there are at least eight newer FQs on the market [3], not mentioned here, for which data on both ecotoxicity and presence in the freshwater environment are not yet available. It is hoped that data will also be collected for these more modern FQs in the coming years, to verify whether their environmental impact is lower than that of their predecessors. It will also be essential to perform ecotoxicity tests on different benthic organisms to better assess the risk that FQs pose to the aquatic environment; in this context, their ability to cause delayed, multigenerational, and transgenerational toxic effects in crustaceans should not be overlooked. Finally, future research should also focus on new cost-effective techniques to improve the efficiency of sewage treatment plants in removing FQs.

## Figures and Tables

**Table 1:** EC50s (mg L^−1^) reported for FQs in different taxa with their relative reference. Details on test duration, endpoint, and measured parameters are also provided.

Taxa/Species	Fluoroquinolones	EC_50_	Duration	Endpoint	Parameter	Reference
***Cyanobacteria***						
*Anabaena flos-aquae*	ciprofloxacin	0.0102	72 h	Yield inhibition	Cells number	Ebert et al., 2011 [243]
	enrofloxacin	0.173	72 h	Yield inhibition	Cells number	Ebert et al., 2011 [243]
(*Anabaena* CPB4337)	levofloxacin	4.8	72 h	Reduced luminescence	Luminescence	González-Pleiter et al., 2013 [244]
	norfloxacin	5.6	72 h	Reduced luminescence	Luminescence	González-Pleiter et al., 2013 [244]
*Microcystis aeruginosa*	ciprofloxacin	0.017	5 d	Growth rate inhibition	Fluorescence	Robinson et al., 2005 [245]
		0.005	72 h	Growth rate inhibition	Absorbance	Halling-Sorensen et al., 2000 [246]
	clinafloxacin	0.103	5 d	Growth rate inhibition	Fluorescence	Robinson et al., 2005 [245]
	enrofloxacin	0.049	5 d	Growth rate inhibition	Fluorescence	Robinson et al., 2005 [245]
	flumequine	1.960	5 d	Growth rate inhibition	Fluorescence	Robinson et al., 2005 [245]
			7 d	Growth rate inhibition	Fluorescence	Lützhøft et al., 1999 [247]
	gatifloxacin	0.02530	96 h	Growth rate inhibition	Fluorescence	Wan et al., 2021 [248]
	levofloxacin	0.0079	5 d	Growth rate inhibition	Fluorescence	Robinson et al., 2005 [245]
	lomefloxacin	0.186	5 d	Growth rate inhibition	Fluorescence	Robinson et al., 2005 [245]
	moxifloxacin	0.06034	96 h	Growth rate inhibition	Fluorescence	Wan et al., 2021 [248]
	norfloxacin	0.03479	72 h	Growth rate inhibition	Fluorescence	Zhao et al., 2021 [249]
	ofloxacin	0.021	5 d	Growth rate inhibition	Fluorescence	Robinson et al., 2005 [245]
	sarafloxacin	0.015	7 d	Growth rate inhibition	Fluorescence	Lützhøft et al., 1999 [247]
*Microcystis panniformis*	ciprofloxacin	0.01356	96 h	Growth rate inhibition	Cells number	Azevedo et al., 2019 [171]
**Unicellular green algae**						
*Raphidocelis subcapitata*	ciprofloxacin	11.3	72 h	Growth rate inhibition	Absorbance	Magdaleno et al., 2015 [250]
		2.97	72 h	Growth rate inhibition	Absorbance	Halling-Sorensen et al., 2000 [246]
		4.83	96 h	Growth rate inhibition	Cells number	Martins et al., 2012 [251]
		18.7	72 h	Growth rate inhibition	Fluorescence	Robinson et al., 2005 [245]
		6.7	72 h	Yield inhibition	Absorbance	Yang et al., 2008 [252]
		7.082	96 h	Growth rate inhibition	Absorbance	Fu et al., 2017 [253]
	clinafloxacin	1.1	72 h	Growth rate inhibition	Fluorescence	Robinson et al., 2005 [245]
	enrofloxacin	3.1	72 h	Growth rate inhibition	Fluorescence	Robinson et al., 2005 [245]
	flumequine	5	72 h	Growth rate inhibition	Fluorescence	Robinson et al., 2005 [245]
		5	72 h	Growth rate inhibition	Fluorescence	Lützhøft et al., 1999 [247]
		2.6	96 h	Growth rate inhibition	Absorbance	Zounková et al., 2011 [254]
		16	24 h	Yield inhibition	Fluorescence	Van Der Grinten et al., 2010 [255]
		8.1	48 h	Growth rate inhibition	Fluorescence	Christensen et al., 2006 [256]
	levofloxacin	7.4	72 h	Growth rate inhibition	Fluorescence	Robinson et al., 2005 [245]
		1.2	96 h	Growth rate inhibition	Absorbance	Yamashita et al., 2006 [257]
	lomefloxacin	22.7	72 h	Growth rate inhibition	Fluorescence	Robinson et al., 2005 [245]
	norfloxacin	16.6	72 h	Growth rate inhibition	Cells count	Eguchi et al., 2004 [258]
		18	72 h	Yield inhibition	Absorbance	Yang et al., 2008 [252]
		59.404	96 h	Growth rate inhibition	Absorbance	Fu et al., 2017 [253]
	ofloxacin	12.1	72 h	Growth rate inhibition	Fluorescence	Robinson et al., 2005 [245]
		4.241	96 h	Growth rate inhibition	Absorbance	Fu et al., 2017 [253]
		1.44	72 h	Growth rate inhibition	Cells number	Isidori et al., 2005 [259]
		4.74	96 h	Growth rate inhibition	Cells number	Ferrari et al., 2004 [260]
	sarafloxacin	16	72 h	Growth rate inhibition	Fluorescence	Lützhøft et al., 1999 [247]
*Chlamydomonas reinhardtii*	gatifloxacin	83.04	7 d	Growth rate inhibition	Absorbance	Wan et al., 2022 [261]
	moxifloxacin	12.65	7 d	Growth rate inhibition	Absorbance	Wan et al., 2022 [261]
*Chlorella vulgaris*	ciprofloxacin	29.09	96 h	Growth rate inhibition	Absorbance	Geiger et al., 2016 [262]
		20.6	96 h	Growth rate inhibition	Cells number	Nie et al., 2008 [263]
	norfloxacin	10.4	72 h	Growth rate inhibition	Cells count	Eguchi et al., 2004 [258]
*Chlorella sorokiniana*	moxifloxacin	28	96 h	Growth rate inhibition	Absorbance	Li et al., 2023 [264]
*Chlorella sp.*	ciprofloxacin	23	72 h	Growth rate inhibition	Cells number	Andrieu et al., 2015 [53]
	enrofloxacin	111	72 h	Growth rate inhibition	Cells number	Andrieu et al., 2015 [53]
*Desmodesmus subspicatus*	enrofloxacin	5.568	72 h	Yield inhibition	Cells number	Ebert et al., 2011 [243]
*Scenedesmus vacuolatus*	flumequine	3.7	24 h	Growth rate inhibition	Cells number	Backhaus et al., 2001 [265]
	lomefloxacin	58	24 h	Growth rate inhibition	Cells number	Backhaus et al., 2001 [265]
	norfloxacin	69.6	24 h	Growth rate inhibition	Cells number	Backhaus et al., 2001 [265]
	ofloxacin	82.8	24 h	Growth rate inhibition	Cells number	Backhaus et al., 2001 [265]
*Scenedesmus obliquus*	enrofloxacin	45.10	96 h	Growth rate inhibition	Absorbance	Qin et al., 2012 [266]
	norfloxacin	38.49	96 h	Growth rate inhibition	Cells number	Nie et al., 2009 [267]
		50.18	96 h	Growth rate inhibition	Cells number	Lu et al., 2007 [268]
*Scenedesmus dimorphus*	moxifloxacin	26	96 h	Growth rate inhibition	Absorbance	Li et al., 2023 [264]
**Aquatic plants**						
*Lemna minor*	ciprofloxacin	0.0625	7 d	Yield inhibition	Dry weight	Ebert et al., 2011 [243]
		0.203	7 d	Growth rate inhibition	Fronds number	Robinson et al., 2005 [245]
	clinafloxacin	0.062	7 d	Growth rate inhibition	Fronds number	Robinson et al., 2005 [245]
	enrofloxacin	0.107	7 d	Yield inhibition	Dry weight	Ebert et al., 2011 [243]
		0.114	7 d	Growth rate inhibition	Fronds number	Robinson et al., 2005 [245]
	flumequine	2.470	7 d	Growth rate inhibition	Fronds number	Robinson et al., 2005 [245]
		3.0	7 d	Yield inhibition	Fronds number	Zounková et al., 2011 [254]
	levofloxacin	0.051	7 d	Growth rate inhibition	Fronds number	Robinson et al., 2005 [245]
	lomefloxacin	0.106	7 d	Growth rate inhibition	Fronds number	Robinson et al., 2005 [245]
	ofloxacin	0.126	7 d	Growth rate inhibition	Fronds number	Robinson et al., 2005 [245]
*Lemna gibba*	ciprofloxacin	0.698	7 d	Yield inhibition	Wet weight	Brain et al., 2004 [269]
	levofloxacin	0.532	7 d	Yield inhibition	Wet weight	Brain et al., 2004 [269]
	lomefloxacin	0.185	7 d	Yield inhibition	Wet weight	Brain et al., 2004 [269]
	norfloxacin	0.913	7 d	Yield inhibition	Fronds number	Brooks et al., 2008 [270]
	ofloxacin	0.532	7 d	Yield inhibition	Wet weight	Brain et al., 2004 [269]
**Crustaceans**						
*Daphnia magna*	ciprofloxacin	7.2	48 h	Immobilization	Imm. number	Eluk et al., 2021 [271]
		36.493	48 h	Immobilization	Imm. number	Dionísio et al., 2020 [272]
		87.14	48 h	Immobilization	Imm. number	Dalla Bona et al., 2014 [273]
	enrofloxacin	7.9	48 h	Immobilization	Imm. number	Eluk et al., 2021 [271]
		16.72	48 h	Immobilization	Imm. number	Tolosi & De Liguoro 2021 [21]
		3.13	48 h + 10 d	Delayed immobilization	Imm. number	Tolosi & De Liguoro 2021 [21]
		16.34	48 h	Immobilization	Imm. number	Dalla Bona et al., 2014 [273]
	flumequine	7.18	48 h + 10 d	Delayed immobilization	Imm. number	Tolosi & De Liguoro 2021 [21]
	gatifloxacin	330.8	48 h	Immobilization	Imm. number	Mala & Dutta 2019 [274]
	gemifloxacin	489.2	48 h	Immobilization	Imm. number	Mala & Dutta 2019 [274]
	levofloxacin	28	48 h	Immobilization	Imm. number	Kergaravat et al., 2021 [275]
		19.5	48 h	Immobilization	Imm. number	Eluk et al., 2021 [271]
		15.11	48 h	Delayed immobilization	Imm. number	Tolosi & De Liguoro 2021 [21]
	lomefloxacin	166	48 h	Immobilization	Imm. number	Luo et al., 2018 [276]
	marbofloxacin	5.4	48 h	Immobilization	Imm. number	Eluk et al., 2021 [271]
	moxifloxacin	14	48 h	Immobilization	Imm. number	Kergaravat et al., 2021 [275]
	norfloxacin	8.7	48 h	Immobilization	Imm. number	Eluk et al., 2021 [271]
	ofloxacin	31.75	48 h	Immobilization	Imm. number	Isidori et al., 2005 [259]
		36	48 h	Immobilization	Imm. number	Eluk et al., 2021 [271]
*Daphnia curvirostris*	ciprofloxacin	4.45	48 h	Immobilization	Imm. number	Dalla Bona et al., 2014 [273]
	enrofloxacin	4.33	48 h	Immobilization	Imm. number	Dalla Bona et al., 2014 [273]
*Ceriodaphnia dubia*	ciprofloxacin	36	48 h	Immobilization	Imm. number	Kergaravat et al., 2021 [275]
	enrofloxacin	60	48 h	Immobilization	Imm. number	Kergaravat et al., 2021 [275]
	levofloxacin	35	48 h	Immobilization	Imm. number	Kergaravat et al., 2021 [275]
	marbofloxacin	31	48 h	Immobilization	Imm. number	Kergaravat et al., 2021 [275]
	moxifloxacin	29	48 h	Immobilization	Imm. number	Kergaravat et al., 2021 [275]
	norfloxacin	73	48 h	Immobilization	Imm. number	Kergaravat et al., 2021 [275]
	ofloxacin	17.41	7 d	Immobilization	Imm. number	Isidori et al., 2005 [259]
*Moina macrocopa*	ciprofloxacin	71.2	48 h	Immobilization	Imm. number	Andrieu et al., 2015 [53]
	enrofloxacin	69.1	48 h	Immobilization	Imm. number	Andrieu et al., 2015 [53]
**Fish**						
*Danio rerio*	ciprofloxacin	620	66 h	Embryonic mortality	Dead number	Han et al., 2021 [277]
	enrofloxacin	150	96 h	Embryonic mortality	Dead number	Özhan Turhan 2021 [278]
	flumequine	40	48 h	Embryonic mortality	Dead number	Lancieri et al., 2002 [279]
	gatifloxacin	4393	66 h	Embryonic mortality	Dead number	Han et al., 2021 [277]
	levofloxacin	5437	66 h	Embryonic mortality	Dead number	Han et al., 2021 [277]
	lomefloxacin	1430	66 h	Embryonic mortality	Dead number	Han et al., 2021 [277]
	moxifloxacin	609	66 h	Embryonic mortality	Dead number	Han et al., 2021 [277]
	norfloxacin	1311	66 h	Embryonic mortality	Dead number	Han et al., 2021 [277]

**Table 2:** EC_50_ value of eleven fluoroquinolones expressed as *Microcystis*-equivalents and used for the construction of the normalized Species Sensitivity Distribution curve. Values in brackets represent the number of experimental data available in the literature. Cip: ciprofloxacin; Cli: clinafloxacin; Enr: enrofloxacin; Flu: flumequine; Gat: gatifloxacin; Lev: levofloxacin; Lom: lomefloxacin; Mox: moxifloxacin; Nor: norfloxacin; Ofl: ofloxacin; Sar: sarafloxacin; Mean: geometric mean. Data are obtained from [53,243,244,245,246,247,249,250,251,252,253,254,255,256,257,258,259,260,262,263,266,267,268,269,270,310].

	Cip	Cli	Enr	Flu	Gat	Lev	Lom	Mox	Nor	Ofl	Sar	Mean
*Chlorella* sp.	2600.58 (3)		2265.31 (1)					464.01	397.59			1021.03
*Scenedesmus obliquus*			920.41 (1)						976.62			948.10
*Chlamydomonas* *reinhardtii*					3282.21 (1)			209				828.21
*Scenedesmus* *dimorphus*								430.89				430.89
*Scenedesmus* *vacuolatus*				6.63 (1)			311.83		1546.67	3942.86		335.09
*Raphidocelis* *subcapitata*	744.7 (7)	68.57 (1)	63.27 (1)	10.92 (5)		372.49 (2)	122.04		513	206.01	1066.67	178.16
*Desmodesmus* *subspicatus*			113.63 (1)									113.63
*Anabaena* *flos-aquae*	1.08 (1)		3.53 (1)			600.00 (1)			124.44			23.10
*Lemna gibba*	75.71 (1)					66.50 (1)	0.52		20.29	33.24		17.76
*Lemna minor*	39.41 (3)	6.00 (1)	2.25 (2)	4.88 (2)		6.38 (1)	0.57			6		4.78
*Microcystis* *panniformis*	1.47 (1)											1.47
*Microcystis* *aeruginosa*	1 (2)	1 (1)	1 (1)	1 (2)	1 (1)	1 (1)	1 (1)	1 (1)	1 (1)	1 (1)	1 (1)	1.00

## Data Availability

No new data were created or analyzed in this study. Data sharing is not applicable to this article.
